# Numerical Solution of Magnetized Williamson Nanofluid Flow over an Exponentially Stretching Permeable Surface with Temperature Dependent Viscosity and Thermal Conductivity

**DOI:** 10.3390/nano12203661

**Published:** 2022-10-18

**Authors:** Muhammad Amjad, Iftikhar Ahmed, Kamran Ahmed, Marei Saeed Alqarni, Tanvir Akbar, Taseer Muhammad

**Affiliations:** 1 Department of Mathematics, COMSATS University Islamabad, Islamabad 45550, Pakistan; 2 Department of Mathematics, College of Science, King Khalid University, Abha 61413, Saudi Arabia

**Keywords:** Williamson fluid, MHD, variable thermal conductivity and viscosity, nanofluids, exponential stretching

## Abstract

This research work describes and investigates Williamson nanofluid flow over an exponentially stretching permeable vertical plate with temperature-dependent thermal conductivity and viscosity. The governing non-linear partial differential equations (PDEs) are metamorphosed into coupled non-linear ordinary differential equations (ODEs) by using similarity transformation. The succeeding equations were numerically solved using MATLAB function bvp4c for various values of parameters. For velocity, temperature, concentration, the skin friction coefficient, and the local Nusselt number, data are presented in the form of graphs and tables. It is noted that for increasing values of magnetic parameter M, Williamson parameter λ, and viscosity parameter α, the boundary layer thickness of the velocity profile decreases, while it increases for the temperature profile. The findings of the present work are validated through the published results.

## 1. Introduction

The research on non-Newtonian fluids has received attention because of the limited application of Newtonian fluids. Honey, ketchup, lubrication sprays, and starch, etc., are the examples of non-Newtonian fluids. Williamson [1] presented a non-Newtonian model for fluids in 1929, which depicts the rheological properties of such fluids. It is known as the Williamson fluid model in literature. Nadeem et al. [2] examined the heat transfer impacts for magnetized Williamson fluid over an exponentially stretching sheet. Amjad et al. [3] studied the MHD Williamson nanofluid with the Cattaneo–Christov (CC) heat flux model across an exponential stretching sheet. As a result of its usefulness, several researchers [4,5,6,7,8,9,10] have used the Williamson model to show the true behavior of fluids throughout the previous decade.

Magnetohydrodynamics (MHD) theory is attributed to the famous Nobel laureate Hannes Alfven. It is a mix of fluid mechanics and electromagnetism, which describes the behavior or treatment of a magnetic field on an electrically conducting fluid. Cancer tumor therapy, blood flow, cell separation, centrifugal pumps, tissue temperatures, and magnetic endoscopy are the applications of magnetohydrodynamics. Makinde et al. [11] studied 2D boundary layer flow of nanofluids which are chemically reacting with thermal radiation across a non-linear stretched plate. Turkyilmazoglu [12] used an analytical strategy to cope with the MHD flow. Mabood et al. [13] studied the magnetized flow of a spinning fluid along a vertical plane. Talihnoee et al. [14] explored the creation of MHD entropy and spontaneous convection in nanofluids. Rashidi et al. [15] proposed that the MHD Williamson fluid has heat radiation and thermodiffusion effects on stretching porous sheets. Several researchers have recently focused on MHD fluid flows due to their extensive range of application such as engineering and industry [16,17,18,19,20].

The traditional heat transfer fluids with low thermal conductivity, such as oil and water, are not able to meet the increasing demand of the more sophisticated heat transfer technologies. To resolve this issue, small solid nanoparticles are added to boost the heat conductivity of these convectional fluids. As a result, nanofluids are fluids formed by suspending tiny volumes of nanometer-sized particles in standard liquids. Choi [21] was the first to investigate and improve the thermal conductivity of fluids. Azam et al. [22,23,24,25,26] analyzed the impacts of Cattaneo–Christov (CC) heat flux, viscous dissipation, and transient bioconvection on magnetized Maxwell and Casson nanofluid. Azam et al. [25,26] also studied the bioconvection, activation energy dynamisms, and nonlinear thermal extrusion with gyrotactic microorganism on radiative sutterby melting nanomaterial. Sahu et al. [27] studied the Darcy–Forchheimer flow behavior and thermal inferences due to shrinking rotating disk with SW/MWCNT suspensions. Many authors have recently investigated numerous boundary layer movement challenges of a nanofluid with varied geometries [28,29,30,31,32].

Thermal conductivity is an important characteristic of a substance in the heat transmission procedure since it monitors the heat transfer rate in manufactured goods that rely on heat-regulating elements and high-level temperature. Many researchers [33,34,35,36,37] have been fascinated by the impact of changing heat conductivity and viscosity on thin film flows, nanofluid flows, and dusty fluid flows. As a result, Salawu et al. [38] analyzed the impacts of changing viscosity on radiative heat transfer and thermal conductivity by using magnetic field vertically. MHD flow with variable thermal conductivity and viscosity with a point source was studied by Choudhury et al. [39]. Shojaeian et al. [40] explored fast heat transmission and second-law assessment of non-Newtonian liquid flow in circular channels with changing thermophysical characteristics.

In the present model, we considered an exponential stretching sheet with temperature-dependent viscosity and thermal conducting using a permeable vertical surface, which has not been considered in previous models. Using similarity transformations, the system of non-linear PDEs that governs the model are transformed into a non-linear ODEs. The built-in MATLAB function bvp4c is used to solve the non-linear set of ODEs. Tables and graphs are presented to understand the impact of numerous physical parameters. To the best of our knowledge and understanding, no prior research of this kind has been undertaken.

## 2. Problem Description

**Figure 1:**
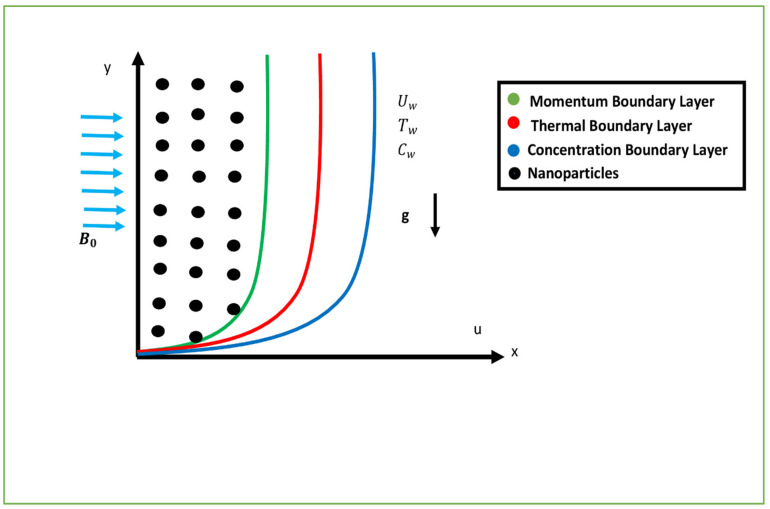
Schematic geometry of the flow problem. $U_{w}$ stands for the velocity at the wall, $T_{w}$ denotes the wall’s temperature, $C_{w}$ denotes the concentration at the wall, g denotes the gravitational acceleration, and $B_{0}$ denotes the applied magnetic field.

Consider a steady and incompressible two-dimensional magnetized Williamson nanofluid flow flowing over an exponentially stretching porous surface. The surface is taken vertically. The plate is taken along the x− axis and the y− axis is perpendicular to it. The fluid is flowing with velocity $U_{w}$ and is perpendicular to the plane y≥0. The transverse magnetic field $B=B_{0}e^{\frac{x}{2l}}$ affects the flow direction perpendicularly. The physical model is systematically shown in Figure 1. The Stress-tensor ($C_{S})$ for the Williamson nanofluid model, as reported by [40,41], is represented by:

(1) $$C_{S}=-pI+\tau ,$$

(2) $$\tau =\mu_{\infty }+\left(\frac{\mu_{0}-\mu_{\infty }}{1-\Gamma \;\dot{\gamma }}\right)A_{1},$$

where $C_{s}$ $\mu_{\infty },$ $\mu_{0}$, Γ>0, and $A_{1}$ represent additional stress tensor, infinite and zero-shear rate limiting viscosity, time constant and first Rivlin–Erickson-tensor. $\dot{\gamma }$ is described below:

(3) $$\dot{\gamma \;}=\sqrt{\frac{\pi }{2}},$$

(4) $$tace{\left(A_{1}\right)}^{2}=2{\left(\frac{\partial u}{\partial y}\right)}^{2}=\pi ,$$

Suppose $\mu_{\infty }=0$ and $\Gamma \;\dot{\;\gamma <1}$. As a result, τ may be written as:

(5) $$\tau =\left(\frac{\mu_{0}}{1-\Gamma \dot{\gamma }}\right)A_{1},$$

We obtain the following results via binomial expansion:

(6) $$\tau =\left(\mu_{0}+\left(1+\Gamma \dot{\gamma }\right)\right)A_{1},$$

The governing Equations (7)–(10) derived in [6,42,43,44,45] for the under-consideration problem are:

(7) $$\frac{\partial u}{\partial x}+\frac{\partial v}{\partial y}=0,$$

(8) $$\rho \left(v\frac{\partial u}{\partial y}+u\frac{\partial u}{\partial x}\right)=\frac{\partial }{\partial y}\left(\mu \left(T\right)\frac{\partial u}{\partial y}+\mu \left(T\right)\sqrt{2}\Gamma {\left(\frac{\partial u}{\partial y}\right)}^{2}\right)-\sigma B_{0}^{2}u-\frac{\mu }{k_{0}}u\;+\rho g\beta_{T}\left(T-T_{\infty }\right)+\rho g\beta_{c}\left(C-C_{\infty }\right),$$

(9) $$\rho c_{p}\left(u\frac{\partial T}{\partial x}+v\frac{\partial T}{\partial y}\right)=\frac{\partial }{\partial y}\left(k\left(T\right)\frac{\partial T}{\partial y}\right)+\rho c_{p}\left[D_{B}\frac{\partial C}{\partial y}\frac{\partial T}{\partial y}+\frac{D_{T}}{T_{\infty }}{\left(\frac{\partial T}{\partial y}\right)}^{2}\right]\;\;+\sigma B_{0}^{2}u^{2}+q^{*},$$

(10) $$u\frac{\partial C}{\partial x}+v\frac{\partial C}{\partial y}=\frac{D_{T}}{T_{\infty }}\frac{\partial^{2}T}{\partial y^{2}}+D_{B}\frac{\partial^{2}C}{\partial y^{2}},$$

where q* represents the non-uniform heat sink or source given as:

(11) $$q^{*}=\frac{k\left(T\right)U_{w}}{2l\nu }\left(D_{1}\left(T_{w}-T_{\infty }\right)f^{\prime }+E_{1}\left(T-T_{\infty }\right)\right),$$

(12) $$k\left(T\right)=k_{\infty }\left(1+\epsilon \theta \left(\eta \right)\right),\;\mu \left(T\right)=\mu_{\infty }e^{-\alpha \theta }.$$

Here, u,v represent the x-component and y-component of the velocity, respectively, in cartesian coordinates, ν, σ, $B_{0}$, Γ, $D_{T}$, $D_{B}$, $c_{p}$, ρ, $k\left(T\right)$, ϵ, $k_{\infty }$, $U_{w}$, $T_{\infty }$, $C_{\infty }$, $T_{w}$, $C_{w}$, α, $\mu \left(T\right)$, $\beta_{C}$, $\beta_{T}$, and g are kinematic viscosity, electrical conductivity, constant magnetic field, time constant, coefficient of thermophoresis diffusion, specific heat, density, Brownian diffusivity, variable thermal conductivity, small parameter, ambient thermal conductivity, wall velocity, ambient temperature and concentration, wall temperature and concentration, dynamic viscosity, variable viscosity, concentration expansion coefficient, thermal expansion coefficient, gravitational acceleration, respectively. In the above, $D_{1}>0$, $E_{1}>0$ corresponds to internal heat generation, which will increase the temperature, whereas $D_{1}<0$, $E_{1}<0$ corresponds to internal heat absorption, which will decrease the temperature. Additionally, $D_{1}$ and $E_{1}$ represents the space and temperature dependent heat source/sink. The following are the relevant boundary conditions illustrated below:

(13a) $$u\left(x,0\right)=U_{w}=U_{0}e^{\frac{x}{l}},\;\;\;\;\;\;\;\;\;\;\;\;\;\;\;\;\;\;\;\;\;\;\;\;\;\;\;\;\;\;\;\;\;\;\;\;\;\;\;\;\;\;\;\;v\left(x,0\right)=-\gamma \left(x\right),$$

(13b) $$T\left(x,0\right)=T_{w},\;\;\;\;\;\;\;\;\;\;\;\;\;\;\;\;\;\;\;\;\;\;\;\;\;\;\;\;\;\;\;\;\;\;\;\;\;\;\;\;\;\;\;\;\;\;\;\;\;\;\;\;\;C\left(x,0\right)=C_{w},$$

(13c) $$u\left(x,\infty \right)=0,\;\;\;T\left(x,\infty \right)=T_{\infty },\;\;\;\;\;\;\;\;\;\;\;\;\;\;\;\;\;\;\;\;\;\;C\left(x,\infty \right)=C_{\infty },$$

where $U_{w}=U_{0}e^{\frac{x}{l}},\;\gamma \left(x\right)=-V_{0}e^{\frac{x}{2l}}$.

The similarity transformations are utilized to solve the Equations (8)–(10).

(14a) $$u=U_{0}e^{\frac{x}{l}}f^{\prime }\left(\eta \right),\;\;\;\;\;\;\;\;\;v=-\sqrt{\frac{U_{0}\nu }{2l}}e^{\frac{x}{2l}}\left(f\left(\eta \right)+\eta f^{\prime }\left(\eta \right)\right),$$

(14b) $$\psi =\sqrt{2U_{0}\nu l}e^{\frac{x}{2l}}f,\;\;\;\;\;\;\;\;\;\;\;\;\;\;\;\;\;\;\;\;\;\;\;\;\eta =\sqrt{\frac{U_{0}}{2\nu l}}\;ye^{\frac{x}{2l}}$$

(14c) $$g=\frac{C-C_{\infty }}{C_{w}-C_{\infty }},\;\;\;\;\;\;\;\;\;\;\;\;\;\;\;\;\;\;\;\;\;\;\;\;\;\;\;\;\;\;\;\;\;\;\theta =\frac{T-T_{\infty }}{T_{w-}T_{\infty }}.$$

The continuity equation is fulfilled, and the result of Equation (8) is given as:

(15) $$e^{-\alpha \theta }\left(\left(1+\lambda f^{\prime \prime }\right)f^{\prime \prime \prime }-\alpha \theta^{\prime }f^{\prime \prime }\left(1+\frac{\lambda }{2}f^{\prime \prime }\right)\;\right)-2{f^{\prime }}^{2}+ff^{\prime \prime }-Mf^{\prime }\;-\Upsilon f^{\prime }+Gr_{T}\theta +Gr_{C}g=0$$

(16) $$f\left(0\right)=S,f^{\prime }\left(0\right)=1,f^{\prime }\left(\infty \right)\to 0.$$

Using Equation (14) in Equations (9) and (10), we arrived at

(17) $$\begin{array}{cc} \left(1+\epsilon \theta \right)\theta^{\prime \prime }+\epsilon {\theta^{\prime }}^{2}\\ \; & \;+\mathrm{Pr}\left(f\theta^{\prime }-f^{\prime }\theta +N_{b}\;g^{\prime }\;\theta^{\prime }+N_{t}\;\theta^{\prime 2}+MEcf^{\prime 2}\right)\\ \; & \;+\left(1+\epsilon \theta \right)\left(D_{1}f^{\prime }+E_{1}\theta \right)=0, \end{array}$$

(18) $$g^{\prime \prime }+Scfg^{\prime }+\frac{N_{t}}{N_{b}}\theta^{\prime \prime }=0,$$

with the boundary conditions:

(19) $$\theta^{\;}\left(0\right)=1,\;g\left(0\right)=1,\;\theta \left(\infty \right)=0,\;\;g\left(\infty \right)=0.$$

In which, $\lambda =\Gamma \sqrt{\frac{U_{0}{}^{3}e^{\frac{3x}{l}}}{\nu l}}$, $M=\frac{2l\sigma B^{2}}{\rho U_{0}}$, $S=V_{0}\sqrt{\frac{2l}{\nu U_{0}}}$, $\Upsilon =\frac{2\nu le^{-\frac{x}{l}}}{k_{0}U_{0}}$, $Gr_{T}=\frac{2lg\beta_{T}{\left(T_{w}-T_{\infty }\right)}_{\;}}{U_{0}e^{\frac{2x}{l}}},$ $Gr_{C}=\frac{2lg\beta_{C}{\left(C_{w}-C_{\infty }\right)}_{\;}}{U_{0}e^{\frac{2x}{l}}}$, $N_{b}=\frac{\rho_{p}c_{p}}{\rho c}\frac{C_{w}-C_{\infty }}{\nu }D_{B},$ $N_{t}=\frac{\rho_{p}c_{p}}{\rho c}\frac{D_{T}\left(T_{w}-T_{\infty }\right)}{\nu T_{\infty }}e^{\frac{x}{2l}}$, $Pr=\frac{\mu c_{p}}{k_{\infty }}$, $Sc=\frac{\nu }{D_{B}},$ $Ec=\frac{U_{w}{}^{2}}{C_{p}}\left(T_{w}-T_{\infty }\right)$.

Here, M, λ, $Gr_{T}$, Nb $Gr_{C}$, Υ, Nt, S, Pr, Sc, and Ec are the magnetic parameter, Williamson parameter, local Grashof number, Brownian motion parameter, local modified Grashof number, permeability parameter, thermophoresis parameter, suction/injection parameter, Prandtl number, Schmidt number, and Eckert number, respectively.

The $c_{f},\;N_{u_{x}},\;$ and $\;Sh_{x}$ are specified as [6,42,43,44]:

(20) $$c_{f}=\frac{\tau_{w}}{\rho U_{w}{}^{2}},$$

(21) $$N_{u_{x}}=\;\;{\left(\frac{xq_{w}}{k\left(T\right)\left(T_{w}-T_{\infty }\right)}\right)}_{y=0},$$

(22) $$Sh_{x}={\left(\frac{xj_{w}}{D_{B}\left(C_{w}-C_{\infty }\right)}\right)}_{y=0}$$

where $\tau_{w}$, $q_{w}$, and $j_{w}$ are given as:

(23) $$\tau_{w}=\left(\mu \left(T\right)\left(\frac{\partial u}{\partial y}+\frac{Г}{\sqrt{2}}{\left(\frac{\partial u}{\partial y}\right)}^{2}\right)\right){,}_{\;}$$

(24) $$q_{w}=-k\left(T\right)\frac{\partial T}{\partial y},$$

(25) $$j_{w}=-D_{B}\;\frac{\partial C}{\partial y}.$$

Dimensionless forms of $c_{f}$, $N_{u_{x}}$, and $Sh_{x}$ are

(26) $$\sqrt{Re_{x}}c_{f}=-2\left(f^{\prime \prime }\left(0\right)+\frac{\lambda }{2}{f^{\prime \prime }}^{2}\left(0\right)\right)e^{-\alpha \theta }{}_{\;},$$

(27) $$\frac{N_{u_{x}}}{\;\sqrt{Re_{x}}}=-\left(1+\epsilon \theta \left(0\right)\right)\theta^{\prime }\left(0\right),$$

(28) $$\frac{Sh_{x}}{\sqrt{Re_{x}}}=-g^{\prime }\left(0\right).$$

where the Reynolds number $Re_{x}=\frac{U_{w}l}{\nu }$.

## 3. Results and Discussion

The coupled nonlinear ODEs Equations (15), (17) and (18) along with boundary conditions (BCs) Equations (16) and (19) are solved numerically using the MATLAB built-in function bvp4c. In order to find the numerical solution of the coupled ODEs Equations (15), (17) and (18) using bvp4c, we rewrite Equations (15), (17) and (18) as  an equivalent system of first-order nonlinear ODEs using $f=\zeta_{1},f^{\prime }=\zeta_{2},f^{\prime \prime }=\zeta_{3},\theta =\zeta_{4},\theta^{\prime }=\zeta_{5},g=\zeta_{6},g^{\prime }=\zeta_{7}$. We then encode these seven first-order ODEs with the function name “nanoode” and convert the first-order boundary conditions to the equivalent of the function name “nanobc” in MATLAB. The interval of integration is chosen to be 0 to 10 and the relative error tolerance $10^{-6}$ is counted in this analysis. We then call the ‘bvp4c’ function and store the obtained numerical results in the variable called “sol”.


sol = bvp4c (@nanoode, @nanobc, solinit, options):

The effects of λ, S, Gr, M, Υ, Nt, Ec, Nb, Pr, and Sc on the velocity, temperature, and concentration distributions have been investigated comprehensively. In our numerical simulations, we use M=1.5, λ=0.2, $Gr_{T}=0.2$, $Gr_{C}=0.2$, Υ = 0.5, S = 0.1, Nb=0.5, Nt=0.5, Sc=0.6, Ec=0.2, $D_{1}=0.1$, $E_{1}=0.1$, α=0.5, and Pr=2.0. To validate our numerical results obtained using bvp4c in MATLAB, we compare the numerical values of the Nusselt number ($-\theta^{\prime }\left(0\right))\;$ against different values of the Prandtl number with the values reported in [33] and [46,47,48]. These findings are closely related to the results published in the literature [33] and [46,47,48], as displayed in Table 1. 

Table 2 and Table 3 illustrate the influences of numerous physical parameters on the skin-friction ($C_{f}$), Nusselt number ($-\theta^{\prime }\left(0\right))$, and Sherwood number ($-g^{\prime }\left(0\right))$. The numerical findings presented in Table 2 reveals that $C_{f}$ decreases for the increasing values of λ, Gr,  and α, whereas it improves for the increasing values of M, S, and Υ. The $-\theta^{\prime }\left(0\right)$ increases against the rising values of  Gr  and M and declines against the increasing values of λ, S, Υ, and α. The $-g^{\prime }\left(0\right)$ is increased against the rising values of  Gr and increases for the increasing values of M, λ, Gr, and α and it decreases for the increasing values of S, and Υ. The numerical results in Table 3 show that both $-\theta^{\prime }\left(0\right)$ and $C_{f}\;$ decrease, whereas the local Sherwood number increases against the increasing values of Ec, ϵ, $D_{1}$, $E_{1}$, and Nt. Table 3 reveals the increase in the skin friction and the Nusselt number for the increasing values of the Prandtl number, whereas it shows the decrease in the local Sherwood number for the increasing values of the Prandtl number Pr. The local Nusselt number decreases, whereas the $-\theta^{\prime }\left(0\right))$ and the $C_{f}$ increase, for the increasing values of Nb and Sc.

**Figure 2:**
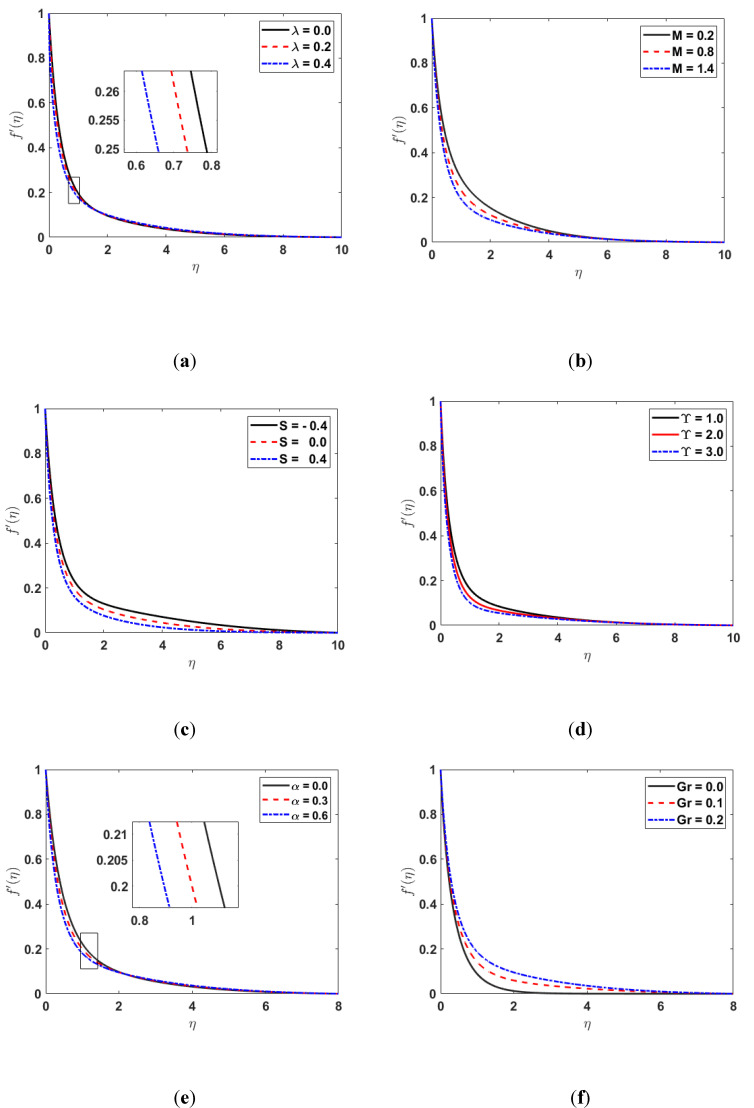
(**a**) $f^{\prime }\left(\eta \right)$ for different values of λ; (**b**) $f^{\prime }\left(\eta \right)\;$ along M; (**c**) $f^{\prime }\left(\eta \right)\;$ along  S; (**d**) $f^{\prime }\left(\eta \right)$ along Υ; (**e**) $f^{\prime }\left(\eta \right)$ along α; (**f**) $f^{\prime }\left(\eta \right)$ along Gr.

Figure 2 displays the influence of λ, M, S, Υ, and Gr on the velocity distribution. Figure 2b depicts that the velocity boundary layer reduces by enhancing the magnetic parameter (M). Since the magnetic field acts as a hurdle to transport the fluid flow, it causes lessening in the velocity profile. Additionally, the rise in M enhances the Lorentz force, which generates resistance to the transport phenomenon; hence, it also decreases the fluid’s velocity. The impact of λ on the velocity profile is shown in Figure 2a. Its effect on the velocity profile is the same as earlier discussed in Figure 2b. The graphs in Figure 2c displays that the $f^{\prime }\left(\eta \right)$ decreases as the suction/injection parameter S increases. Figure 2d demonstrates the influence of the permeability parameter Υ on the velocity profile. In Figure 2d, it can be noticed that the $f^{\prime }\left(\eta \right)$ decreases as the value of the permeability parameter  Υ increases. Figure 2e depicts the effect on the $f^{\prime }\left(\eta \right)\;$ for the various values of the viscosity parameter α. The plots in Figure 2f show that the $f^{\prime }\left(\eta \right)$ increases with the growing values of Gr since the buoyancy effect enhances fluid flow speed.

**Figure 3:**
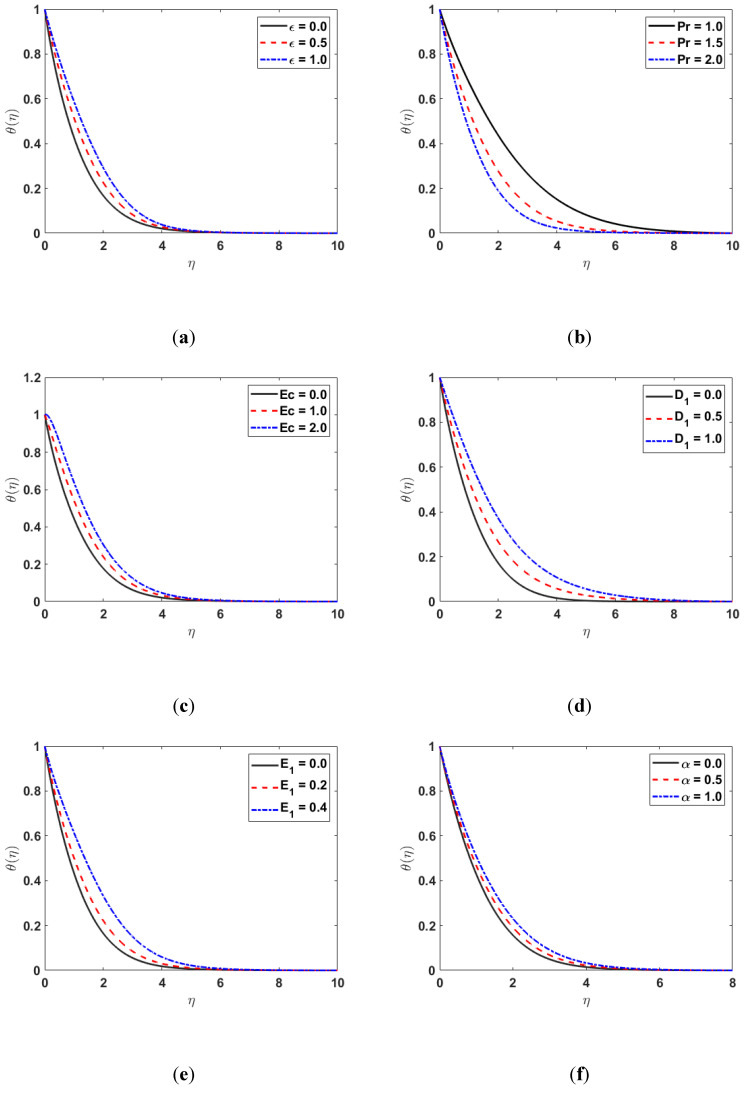
(**a**) $\theta \left(\eta \right)$ along ϵ; (**b**) $\theta \left(\eta \right)$ along Pr; (**c**) $\theta \left(\eta \right)$ along Ec; (**d**) $\theta \left(\eta \right)$ along $D_{1};$ (**e**) $\theta \left(\eta \right)$ along $\;E_{1};$ (**f**) $\theta \left(\eta \right)$ along α.

The effect of the variable thermal conductivity ϵ on the temperature distribution $\theta \left(\eta \right)$ is indicated in Figure 3a. The temperature of the fluid increases by increasing the thermal conductivity ϵ since the transmission of heat from the sheet to the fluid is higher for the higher values of ϵ. Figure 3b characterizes the impact of the Prandtl number Pr on $\theta \left(\eta \right)$. Figure 3b shows that when the Prandtl number Pr increases, the temperature profile $\theta \left(\eta \right)$ decreases. Since the Prandtl number Pr is the ratio of momentum to thermal diffusivity, the temperature profile $\theta \left(\eta \right)$ decreases by increasing Pr. The effect of the Eckert number on $\theta \left(\eta \right)$ is presented in Figure 3c. The graph in Figure 3c shows that raising the Ec will increase in $\theta \left(\eta \right)$. The influence of irregular heat parameters $D_{1}$ and $E_{1}$ on $\theta \left(\eta \right)$ is depicted in Figure 3d,e. The $\theta \left(\eta \right)$ is enhanced as the values of $D_{1}$ and $E_{1}$ are increased. Figure 3f illustrates the impacts of the viscosity parameter α on $\theta \left(\eta \right)$. Figure 3f shows that for large values of α, the thermal boundary-layer thickness also increases.

**Figure 4:**
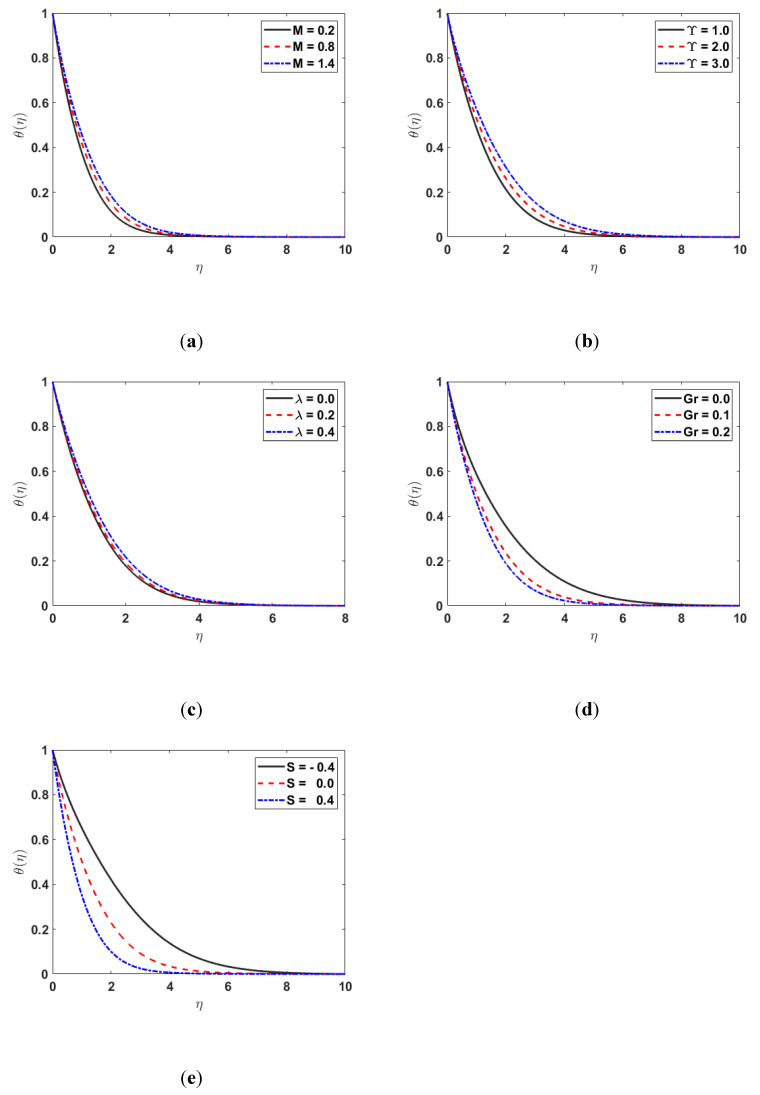
(**a**) θ(η) for different values of M; (**b**) $\theta \left(\eta \right)\;$ for different values of Υ; (**c**) $\theta \left(\eta \right)$ for various values of λ; (**d**) $\theta \left(\eta \right)\;$ along Gr; (**e**) $\theta \left(\eta \right)$ along S.

Figure 4 shows the impact of Υ, λ, M, Gr, and S on $\theta \left(\eta \right)$. Figure 4a illustrates the behavior of M on $\theta \left(\eta \right)$. The fluid’s temperature and the thermal boundary-layer thickness both rise as the magnetic effect rises. Physically, it occurs because the stronger the Lorentz force is, the greater the heat production is from the fluid’s surface, which increases the fluid’s temperature profile. Figure 4b depicts the effect of Υ on $\theta \left(\eta \right)$. Due to an increase in Υ, heat transfer from one particle to another slows down, which is responsible for the decrease in $\theta \left(\eta \right)$. Figure 4c depicts the influence of λ on $\theta \left(\eta \right)$. This has a similar impact to that mentioned earlier in Figure 4a. Figure 4d depicts the influence of Gr on $\theta \left(\eta \right)$. As the Grashoof number enhances, $\theta \left(\eta \right)$ also increases because the buoyancy effect enhances the speed of the fluid, which, as a result, decreases $\theta \left(\eta \right)$. The impact of S on $\theta \left(\eta \right)$ is demonstrated in Figure 4e. It is noted that the temperature profile $\theta \left(\eta \right)$ increases as S increases.

**Figure 5:**
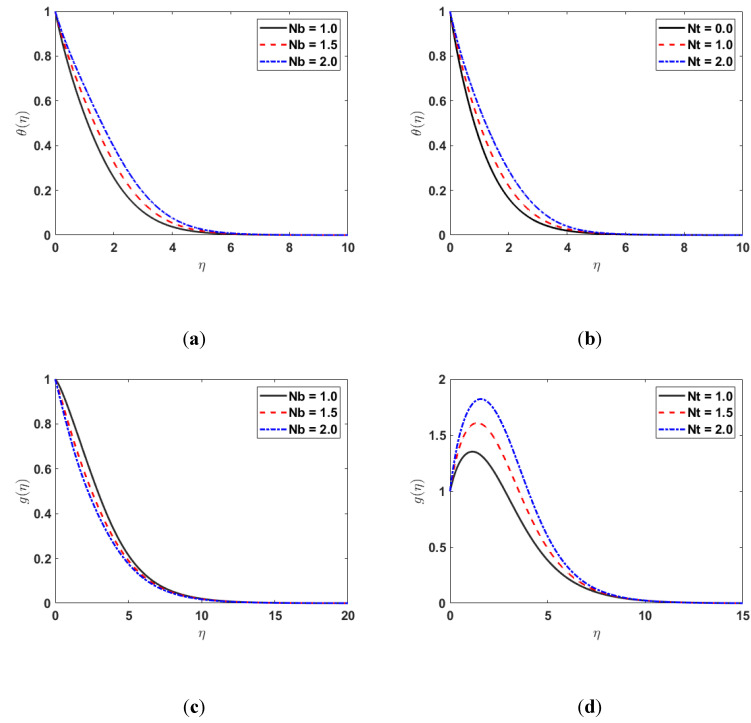
(**a**) $\theta \left(\eta \right)$ along Nb; (**b**) $\theta \left(\eta \right)$ along Nt; (**c**) $g\left(\eta \right)$ along Nb; (**d**) $g\left(\eta \right)$ along Nt.

Figure 5a shows the impact of Nb on $\theta \left(\eta \right)$. Since the temperature profile $\theta \left(\eta \right)$ and Brownian motion parameter Nb are directly linked, the thermal boundary-layer thickness grows with the rising values of Nb. Figure 5b illustrates the effect of Nt on $\theta \left(\eta \right)$. As Nt rises, the temperature profile $\theta \left(\eta \right)$ also rises, thus, the thermal boundary-layer thickness increases. Figure 5c displays the effect of Nb on the concentration profile $g\left(\eta \right)$. By increasing the Nb, the nanofluid concentration $g\left(\eta \right)$ is decreased. The effects of Nt  on the $g\left(\eta \right)$ are depicted in Figure 5d. The graph in Figure 5d shows that when Nt increases, the concentration profile $g\left(\eta \right)$ also increases.

**Figure 6:**
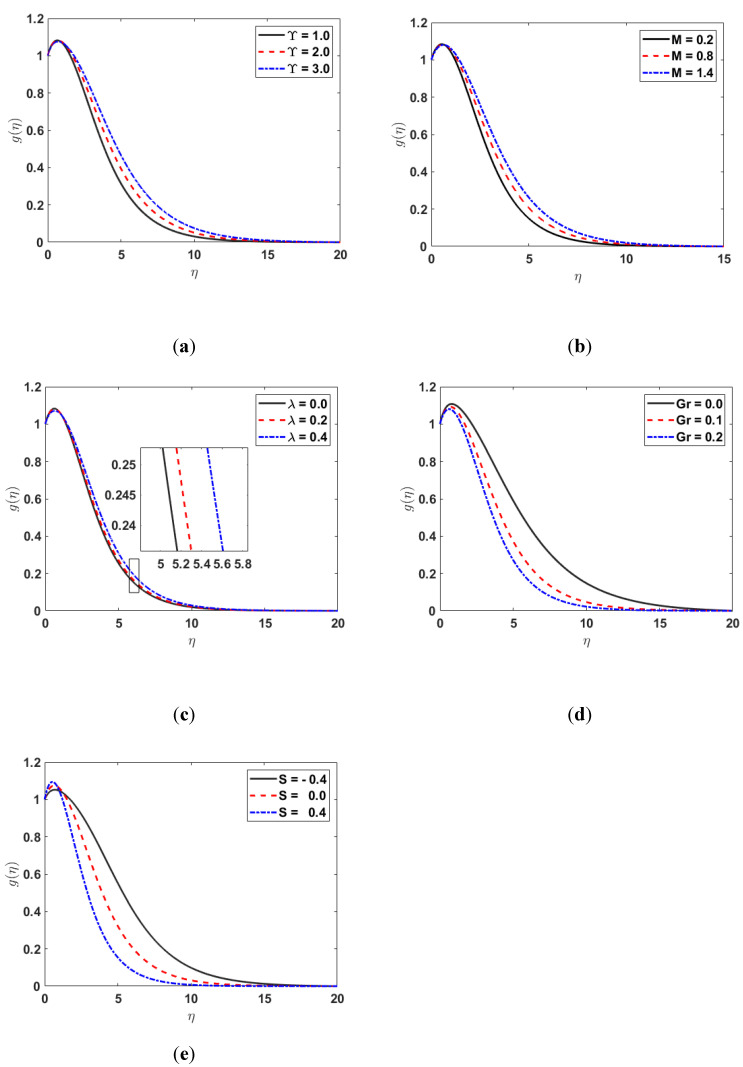
(**a**) $g\left(\eta \right)$ along Υ; (**b**) $g\left(\eta \right)$ along M; (**c**) $g\left(\eta \right)$ along λ; (**d**) $g\left(\eta \right)$ along Gr; (**e**) $g\left(\eta \right)$ along S.

Figure 6 demonstrates the influence of M, Υ, λ, Gr, and S on the concentration-profile $g\left(\eta \right)$. The graphs in Figure 6 show that the $g\left(\eta \right)$ increases for the rising values of Υ, M, and λ, whereas it is reduced for the increasing values of Gr, and S.

**Figure 7:**
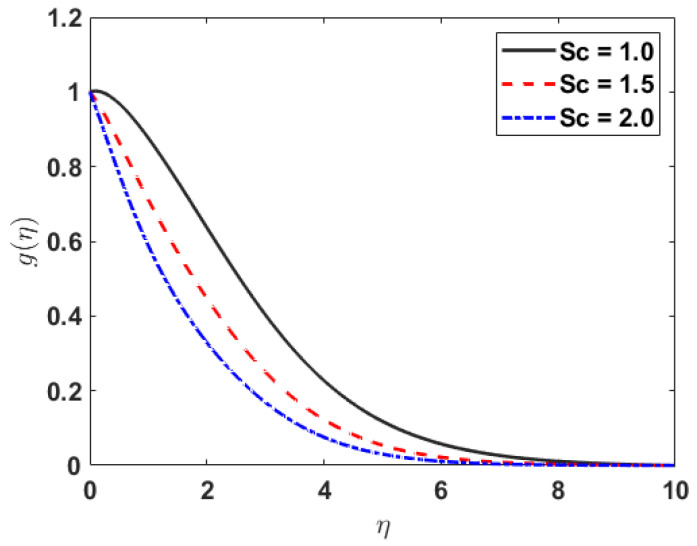
$g\left(\eta \right)$ along Sc.

Figure 7 depicts that the concentration profile $g\left(\eta \right)$e reduces for the growing values of Sc. 

## 4. Conclusions

This research paper provides the numerical solution of the Williamson nanofluid flow across an exponentially stretching, permeable vertical plate with variable temperature-dependent viscosity and thermal conductivity. The following are the significant findings of this study:

- λ, Υ, S, α, and M reduce the velocity profile.
- Increases in M, ε, λ, α, $D_{1}$, $E_{1}$, Ec, Υ, Nb, and Nt enhance the temperature profile $\theta \left(\eta \right)$, whereas increasing values of Gr, S, and Pr decrease the temperature profile $\theta \left(\eta \right)$.
- When M, λ, Υ, and Nt are increased, the concentration profile $g\left(\eta \right)$ also increased, however the concentration profile decreases by increasing the Nb, Gr, S, and Sc.
- $C_{f}$ reduces for the increasing values of ε, λ, α, $D_{1}$, $E_{1}$, Ec, Gr,  and Nt, whereas it grows for the increasing values of M, S, Υ, Nb, Sc, and Pr.
- The temperature profile reduces by increasing the values of M, ε, λ, α, $D_{1}$, $E_{1}$, Ec, Υ, Nb, Sc, and Nt.
- Increasing values of Gr, S, and Pr will increase the Nusselt number.
- The Sherwood number grows for the increasing values of ε, λ, α, M, $D_{1}$, $E_{1}$, Ec, Υ, Nb, Sc, Gr and Nt reduces for the varying values of S and Pr.

## Figures and Tables

**Table 1:** Comparative behavior of $-\theta \prime \left(0\right)$ for different values of Pr when $M=Gr_{T}=Gr_{C}=\Upsilon =S=Ec=D_{1}=E_{1}=\alpha =\lambda =0,\;Nb=0.5$, Nt=0.5.

Pr	Bidin and Nazar [45]	Ishak et al. [33]	Gangaiah et al. [47]	Present
01	0.9547	0.9548	0.9547	0.954810
02	1.4714	1.4715	1.4714	1.471454
03	1.8691	1.8691	1.8691	1.869068

**Table 2:** Numerical values of $c_{f},\;Nu_{x}$ against several values of λ, S, Υ, α, Gr, and M.

λ	M	S	Υ	α	Gr	$\sqrt{Re_{x}}c_{f}$	$\frac{N_{u_{x}}}{\;\sqrt{Re_{x}}}$	$\frac{Sh_{x}}{\sqrt{Re_{x}}}$
0.1	1.5	0.1	0.5	0.5	0.2	2.727752	0.775284	−0.293661
0.2						2.571702	0.761552	−0.287289
0.3						1.526073	0.742947	−0.278067
0.2	0.1					1.998213	0.944614	−0.358783
	0.2					2.046830	0.929656	−0.354155
	0.3					2.094035	0.915051	−0.349443
		0.1				2.571702	0.761552	−0.287289
		0.2				2.660746	0.831095	−0.321924
		0.3				2.752719	0.904827	−0.358963
			0.1	655		2.426609	0.781899	−0.282999
			0.2			2.464228	0.776845	−0.284455
			0.3			2.500928	0.771766	−0.285645
				0.1		3.142260	0.803924	−0.300502
				0.2		2.994002	0.793901	−0.298051
				0.3		2.849485	0.783491	−0.295032
					0.1	2.690235	0.726148	−0.303969
					0.2	2.571702	0.761552	−0.287289
					0.3	2.453274	0.785131	−0.269947

**Table 3:** Values of $c_{f},$ for several values of Ec, ϵ, $D_{1}$, Pr, $E_{1}$, Nt, Nb, and Sc.

Ec	ϵ	$D_{1}$	Pr	$E_{1}$	Nt	Nb	Sc	$\sqrt{Re_{x}}c_{f}$	$\frac{N_{u_{x}}}{\;\sqrt{Re_{x}}}$	$\frac{Sh_{x}}{\sqrt{Re_{x}}}$
0.1	0.2	0.1	2.0	0.1	0.5	0.5	0.1	2.574717	0.808828	−0.332979
0.2	0.2	0.1	2.0	0.1	0.5	0.5	0.1	2.571702	0.761552	−0.287289
0.3	0.2	0.1	2.0	0.1	0.5	0.5	0.1	2.568694	0.714233	−0.241554
0.2	0.1	0.1	2.0	0.1	0.5	0.5	0.1	2.575906	0.812989	−0.336233
0.2	0.2	0.1	2.0	0.1	0.5	0.5	0.1	2.571702	0.761552	−0.287289
0.2	0.3	0.1	2.0	0.1	0.5	0.5	0.1	2.567902	0.716566	−0.244622
0.2	0.2	0.1	2.0	0.1	0.5	0.5	0.1	2.571702	0.761552	−0.287289
0.2	0.2	0.2	2.0	0.1	0.5	0.5	0.1	2.568182	0.721545	−0.249389
0.2	0.2	0.3	2.0	0.1	0.5	0.5	0.1	2.564637	0.681163	−0.211136
0.2	0.2	0.1	1.1	0.1	0.5	0.5	0.1	2.542794	0.464327	−0.008792
0.2	0.2	0.1	1.2	0.1	0.5	0.5	0.1	2.546907	0.505713	−0.047645
0.2	0.2	0.1	1.3	0.1	0.5	0.5	0.1	2.550705	0.544153	−0.083669
0.2	0.2	0.1	2.0	0.1	0.5	0.5	0.1	2.571702	0.761552	−0.287289
0.2	0.2	0.1	2.0	0.2	0.5	0.5	0.1	2.565090	0.693452	−0.223557
0.2	0.2	0.1	2.0	0.3	0.5	0.5	0.1	2.557173	0.614035	−0.149533
0.2	0.2	0.1	2.0	0.1	0.1	0.5	0.1	2.599697	0.813838	0.198771
0.2	0.2	0.1	2.0	0.1	0.2	0.5	0.1	2.592340	0.800852	0.068572
0.2	0.2	0.1	2.0	0.1	0.3	0.5	0.1	2.585221	0.787761	−0.055755
0.2	0.2	0.1	2.0	0.1	0.5	0.1	0.1	2.472216	0.914849	−3.30832
0.2	0.2	0.1	2.0	0.1	0.5	0.2	0.1	2.539176	0.862518	−1.416708
0.2	0.2	0.1	2.0	0.1	0.5	0.3	0.1	2.559441	0.824444	−0.787946
0.2	0.2	0.1	2.0	0.1	0.5	0.5	0.1	2.564745	0.881992	−0.639324
0.2	0.2	0.1	2.0	0.1	0.5	0.5	0.2	2.566273	0.855000	−0.565911
0.2	0.2	0.1	2.0	0.1	0.5	0.5	0.3	2.567733	0.828938	−0.492478

## Data Availability

Not applicable.

## Nomenclature

x,y	Cartesian coordinates $\left[m\right]$
u,v	Velocity components $\left[{\mathrm{ms}}^{-1}\right]$
$U_{0}$	Rate of stretching surface
$\beta_{T}$	Thermal expansion coefficient
$\beta_{C}$	Concentration expansion coefficient
$B_{0}$	Magnetic field strength $\left[{\mathrm{NmA}}^{-1}\right]$
$C_{f}$	Skin friction coefficient
Pr	Prandtl number
M	Magnetic parameter
T	Fluid temperature $\left[K\right]$
$C_{w}$	Concentration of nanoparticles at the surface [mol]
$k\left.\left(T\right.\right)$	Variable thermal conductivity $\left[W/\left(\mathrm{mK}\right)\right]$
C	Concentration of nanoparticles $\left[\mathrm{mol}\right]$
$C_{\infty }$	Ambient concentration of nanoparticles $\left[\mathrm{mol}\right]$
$U_{w}$	Velocity at the wall $\left[{\mathrm{ms}}^{-1}\right]$
S	Suction/injection parameter
$\theta \left(\eta \right)$	Temperature profile
$g\left(\eta \right)$	Concentration profile
$N_{t}$	Thermophoretic parameter
$Nu_{x}$	Local Nusselt number
$Sh_{x}$	Local Sherwood number
$T_{w}$	Surface temperature $\left[K\right]$
$T_{\infty }$	Ambient temperature $\left[K\right]$
f	Dimensionless stream function
g	Nanoparticle volume fraction
Sc	Schmidt number
$D_{B}$	Brownian diffusion coefficient $\left[m^{2}s^{-1}\right]$
$D_{T}$	Thermophoresis diffusion coefficient $\left[m^{2}s^{-1}\right]$
$D_{1}$	Space-dependent heat source/sink
$E_{1}$	Time-dependent heat source/sink
$\mu_{\infty }$	Infinite viscosity $\left[{\mathrm{Nsm}}^{-2}\right]$
$\mu \left(T\right)$	Variable viscosity $\left[{\mathrm{Nsm}}^{-2}\right]$
$\rho_{c}$	Heat capacity of the nanofluid $\left[Jm^{-3}K^{-1}\right]$
$Re_{x}$	Reynold number
M	Magnetic parameter
$f^{\prime }\left(\eta \right)$	Velocity profile
η	Dimensionless similarity variable
σ	Electrical conductivity $\left[Sm^{-1}\right]$
Γ	Positive time constant
α	Thermal diffusivity $\left[m^{-2}s^{-1}\right]$
$\rho c_{p}$	Heat capacity of nanoparticles $\left[Jm^{-3}K^{-1}\right]$
ν	Kinematic viscosity $\left[m^{-2}s^{-1}\right]$
ρ	Density $\left[\mathrm{kg}m^{-3}\right]$
λ	Williamson fluid parameter
ϵ	Dimensionless parameter for variable thermal conductivity
$q^{*}$	Nonuniform heat source/sink [K/S]
$Gr_{T}$	Thermal Grashof number
$Gr_{C}$	Concentrated Grashof number
